# Case Report: Paroxysmal nocturnal hemoglobinuria in a woman heterozygous for G6PD A-

**DOI:** 10.12688/f1000research.4980.2

**Published:** 2014-10-21

**Authors:** Nieves Perdigones, Mariela Morales, Philip Mason, Monica Bessler

**Affiliations:** 1 Division of Hematology, Department of Pediatrics, Abramson Research Center, The Children’s Hospital of Philadelphia, Philadelphia, 19104, USA; 2 Division of Hematology, University of Pennsylvania School of Medicine, Philadelphia, 19104-4318, USA

## Abstract

We describe a case of paroxysmal nocturnal hemoglobinuria (PNH) in a woman who is heterozygous for the glucose-6-phosphate dehydrogenase A-   ( *G6PDA-*) allele. PNH is associated with one or more clones of cells that lack complement inhibition due to loss of function somatic mutations in the *PIGA* gene. *PIGA* encodes the enzyme phosphatidylinositol glycan anchor biosynthesis, class A, which catalyses the first step of glycosylphosphatidylinisotol ( *GPI*)  anchor synthesis. Two GPI anchored red cell surface antigens regulate complement lysis. G6PD catalyses the first step of the pentose phosphate pathway and enzyme variants, frequent in some populations have been selected because they confer resistance to malaria, are associated with hemolysis in the presence of oxidizing agents including several drugs. The patient had suffered a hemolytic attack after taking co-trimoxazole, a drug that precipitates hemolysis in G6PD deficient individuals. Since both *G6PD* and *PIGA* are X-linked we hypothesized that the *PIGA *mutation was on the X-chromosome carrying the *G6PDA*- allele. Investigations showed that in fact the *PIGA* mutation was on the X-chromosome carrying the normal *G6PD B* allele. We speculate that complement activation on *G6PD A- r*ed cells exposed to Bactrim might have triggered complement activation inducing the lysis of *G6PD B* PNH Type II red blood cells or that the patient may have had a PNH clone expressing *G6PDA-* at the time of the hemolytic episode.

## Introduction

In paroxysmal nocturnal hemoglobinuria (PNH) one or more clones of blood cells develops from stem cells that have an acquired mutation in the X-linked *PIGA* gene ^1^. The *PIGA* gene encodes phosphatidylinositol glycan complementation class A, an enzyme that catalyses an early and essential step in glycosylphosphatidylinositol (GPI) anchor synthesis. Thus cells are deficient in all GPI anchored proteins, including CD55 and CD59 which regulate complement activation. PNH usually develops in patients with aplastic anemia (AA) and it is thought that PNH cells have a growth or survival advantage over the AA cells although the mechanism is not known ^2^. PNH cells can be completely deficient in GPI anchored proteins (Type III) or partially deficient due to residual activity of the PIGA protein (Type II), while PNH Type I cells express GPI-linked proteins normally.

Clinically, PNH is characterized by bone marrow failure, thrombosis and intravascular hemolysis. Recently the use of a complement inhibitor, eculizumab has greatly improved the quality of life of PNH patients as it causes a dramatic reduction in the hemolysis and thrombotic episodes, improvement in anemia, with a stabilization of the hemoglobin levels and reduced transfusion requirements ^3^. eculizumab leads to an increase in the number of circulating red blood cells that otherwise are subject to complement-mediated hemolysis ^4^.

Glucose-6-Phosphate Dehydrogenase (G6PD) deficiency is the most common red blood cell enzymopathy and is estimated to affect around 400 million people worldwide ^5^. It is caused by mutations in the X-linked *G6PD* gene which usually lead to an unstable enzyme. G6PD is needed to maintain NADPH and consequently reduced glutathione levels in red blood cells. G6PD-deficient people, mainly males, can be asymptomatic but are subject to episodes of hemolysis when the red blood cells are subjected to oxidative stress caused by infections, certain drugs or in the case of favism, after eating fava beans ^6^. Several polymorphic variants have been described with specific geographical distributions ^7^. In the African population the most common deficient variant is the G6PD A- variant. Compared with normal G6PD, which is called G6PD B, G6PD A- has two amino acid substitutions Val68Met and Asn126Asp ^8^. These are caused by mutations c.202 G->A and c.376A->G respectively. G6PD A- has a frequency of about 10% in Africans and African Americans. G6PD A differs from G6PD B only by the Asn126Asp change and is electrophoretically distinct but with no significant difference in activity. Though milder than other variants such as G6PD Mediterranean found in Italy, Greece and India, G6PDA- is associated with drug induced hemolysis and patients are advised against taking any substances from a list of those known to cause hemolysis. G6PD deficiency usually only affects hemizygous males and homozygous females but heterozygous females can be affected when, for example, biased X-inactivation has led to a predominance of red blood cells expressing the mutant protein ^9^. Here we present a case of an African American woman who was heterozygous for G6PD deficiency and developed PNH, presenting an opportunity to observe the interaction of these two conditions.

## Materials and methods

Peripheral blood from patient CHOP277.01 was obtained after obtaining written informed consent according to the declaration of Helsinki. The Internal Review Board of the Hospital of the University of Pennsylvania approved this study. DNA and RNA were extracted by using QIAamp DNA and RNA Blood mini Kits, respectively, according to manufacturers’ instructions. Blood samples for fluorescent cytometry and electrophoretic analyses were obtained from EDTA tubes and experiments were performed within 2 hours of blood withdrawal.

PCR primers to detect mutations confirming the *G6PD A-* genotype were designed with Primer3 v4.0. (primers for c.202 G->A mutation: forward 5’- agaagaagatctaccccaccatct-3’ and reverse 5’- ctggtacagagggcagaaccag-3’; primers for c.376A->G: forward 5’-catctgtctgtgtgtctgtctgtc-3’ and reverse 5’- ctcatagagtggtgggaggac-3’). Sanger sequencing was done by the Nucleic Acids core facility at CHOP.

The HUMARA assay was performed as previously described ^10^. Briefly, *HhaI* digested and non digested DNA was subjected to PCR amplification of the first exon of the HUMARA locus (containing a CAG repeat) using fluorochrome-coupled primers. Amplification products were then migrated on an ABI PRISM 3100 Automatic Genetic Analyzer (Applied Biosystems). Allele calling and the area under the curve (AUC) were determined using GeneMapper v.4.0 software (Applied Biosystems). The AUC was used to calculate the skewing from X chromosome inactivation (XCI). The XCI ratio of the digested fraction was corrected with that of the undigested fraction to allow for preferential amplification of the smallest allele (i.e., the allele containing less CAG repeats). Skewing is present when the percentage of the predominant allele exceeds 74%. A percentage of predominant allele between 90% and 100% is considered extreme skewing.

Measurements of oxidative stress ROS assay was performed as previously described ^11^. Briefly, red blood cells were incubated with 0.4mM 20-70-dichlorofluorescein diacetate (DCF; Sigma) dissolved in methanol. After incubation at 37ºC for 15 minutes in a humidified atmosphere of 5% CO2 in air, the cells were washed, resuspended in PBS and analyzed by flow cytometry (FACSCalibur; Becton-Dickinson, Immunofluorometry Systems, Mountain View, CA, USA). The mean fluorescence channel (MFC) was calculated by FACSDiva software. The identity of the red cell population was verified by staining with an antibody to glycophorin-A. To determine the presence of GPI proteins, cells were labeled with a phycoerythrin-conjugated anti-CD55 antibody. For our experiment, cells from a non PNH- non G6PD individual served as control. The MFC of cells stained with 0.4mM DCF, is proportional to generation of ROS.

The electrophoretic mobility of the protein was performed in cellogel strips as previously described ^12^. Hemolysates treated with and without acidified serum were run in order to assess differences in mobility of the G6PD enzyme.

## Case report

A 25-year-old African American woman was referred to the Bone Marrow Failure Outpatient Clinic at the Hospital of the University of Pennsylvania for the evaluation and treatment of her PNH.

The past medical history was significant in that at the age of 19 years she presented to the emergency department with cough and dark urine. She was otherwise previously healthy. Family history was only significant for a sister with sickle cell trait. She was diagnosed with Mycoplasma pneumonia and anemia (Hemoglobin 6.9 g/dL (12–16 g/dL), Hematocrit 19.9% (37–47%)). The anemia was determined to be an autoimmune hemolytic anemia (AIHA; LDH 3170 U/L (87–225 U/L), total bilirubin 1.9 mg/dL (0–1.2 g/dL), indirect bilirubin 1.6 mg/dL (0.2–0.7 mg/dL), reticulocyte count 5.2% (0.5–2.1%)) in the setting of positive IgM cold agglutinin antibodies and positive direct Coombs test. The patient was treated with packed red blood cell transfusions and antibiotics and was discharged. As an outpatient, she was started on steroids, and her hemoglobin stabilized between 9 and 10 g/dL; the cold agglutinin and direct Coombs test became negative. The following year, the patient presented on two separate occasions to the emergency department complaining of abdominal pain and dark urine (urine analysis: RBC 1–2, WBC 1–2, hyaline cylinders: none, bacteria, few, squamous epithelia 10–20, dipstick analysis, blood moderate positive). This was interpreted as a urinary tract infections and treated with antibiotics. Her emergency record states that the patient developed hemoglobinuria after being treated with trimethoprim-sulfamethoxazole (co-trimoxazole). Hemogobinuria was associated with lightheadedness and dizziness as well as a mild increase of her liver enzymes (aspartate aminotransferase 77U/L (14–36)). She was therefore screened twice for Glucose-6-Phosphate-Dehydrogenase (G6DP) deficiency, but showed enzyme activity levels within normal limits. At the age of 20-years she became pregnant. She continued to have a picture of hemolytic anemia (hemoglobin range between 9–10 g/dL with a reticulocyte count of 4%, LDH of 555 U/L, total bilirubin range 0.4–0.5 mg/dL, haptoglobin of 2 mg/dL (41–165mg/dL)) but this time, she had negative cold agglutinins and negative direct Coombs test. Her pregnancy was complicated with the worsening of her anemia and development of thrombocytopenia requiring red cell and platelet transfusions. At 34 weeks of gestation she was seen by a hematologist who sent for PNH testing. Flow cytometry revealed a significant population of PNH cells in both red blood and white blood cells (65% of red blood cells and 94% of granulocytes) with a large proportion of red cells with an intermediate expression of CD59 (58%, granulocytes 12%). She was started on anticoagulation with low molecular heparin. She had intermittent episodes of overt hemoglobinuria. At 35 weeks of gestation she was diagnosed with severe hypertension and was admitted for the induction of labor. Her hospital course was complicated with the development of preeclampsia and an acute flair of hemolysis leading to acute renal failure requiring hemodialysis. A healthy baby was delivered by cesarean section. She was vaccinated against meningococcal infection and initiated on eculizumab (Soliris, Alexion Pharmaceuticals) with prophylactic antibiotics for the first 14 days. Her anticoagulation was switched from heparin to warfarin. Her renal functions recovered to normal over the next three months. The patient decided to discontinue eculizumab and warfarin on her own as she thought that this was not beneficial and she continued to have episodes of dark urine.

Three years later, she presented again with left upper abdominal pain, vomiting and blurry vision. She was diagnosed with an acute hemolytic exacerbation of PNH and was admitted. The patient was found to have anemia, leukocytosis and mild transaminitis. A Magnetic Resonace Venogram (MRV) of the abdomen and pelvis was obtained and it showed non-specific perfusional abnormalities throughout the liver. A Computed Tomography (CT) of the abdomen and pelvis revealed several ill-defined low attenuation lesions of the posterior segment of the right hepatic lobe. This was thought to be consistent with liver thrombosis so she was restarted on anticoagulation with warfarin and was referred to us for further evaluation and treatment recommendations and to rule out resistance to C5 inhibitor therapy.

On review of systems, the patient complained of occasional abdominal pain and headaches. On physical exam, she was found to have mildly icteric sclera. Family history was significant for a sister with sickle cell trait. Further questioning revealed that that one of her nephews was diagnosed with G6PD deficiency. The laboratory workup revealed a white blood cell count 4.6 k/uL (4.5–11.0 k/uL), RBC count of 3.8 k/uL (4.2–5.5 k/uL), hemoglobin of 11.8g/dL, hematocrit 35% (37–47%), MCV 91 fl (82–100 fl), platelets 327 k/uL (150–400 k/uL), reticulocytes 24.5%, PTT 27.8 sec (23–36 sec), INR 1.7, D dimer 2.5 ug/mL (0–0.4), bilirubin 1.3 mg/dL, bilirubin direct 0.2 mg/dL (0–0.4 mg/dL), bilirubin indirect 1.1 mg/dL, ANC 2.7 k/uL (1.75–7.59 k/uL), ALC 1.3 k/uL (1.12–4.95 k/uL), G6PD screen normal, LDH 720 U/L. The flow cytometry at this point revealed that 78% of her red blood cells were PNH, 69% were partially deficient for CD59 and 9.1% lacked CD59 completely; 92% of her granulocytes were PNH, 86% were partially deficient for CD59 and 6% lacked the expression of CD59. Due to her history of hepatic vein thrombosis eculizumab was reinitiated at its regular dosing for patients with PNH (600 mg weekly 4x followed by 900 mg every two weeks). In three years of being on eculizumab she had no further relapse of her hemoglobinuria and no evidence of thrombosis.

## Results

**Figure 1.**
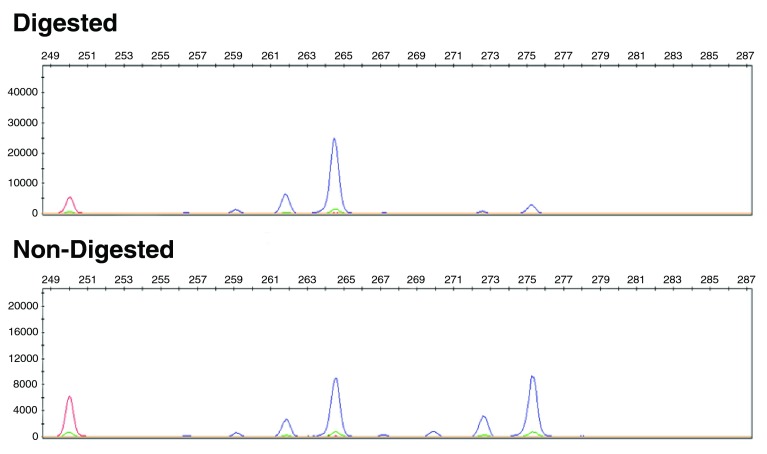
Clonal hematopoiesis in patient CHOP 277.01. The panel at the bottom shows the migration of the 2 microsatellite alleles in patient CHOP277.01 revealed by PCR analysis of a region of the Androgen receptor gene on the X-chromosome. The amplified region contains both a polymorphic repeat and a site for the methylation sensitive restriction enzyme HhaI. This site is methylated on the inactive X-chromosome. The top panel shows the same sample digested with HhaI before the PCR so only the fragment on the inactive X-chromosome is amplified. An imbalance in the allelic ratio reflects an imbalance in X inactivation and therefore indicates clonality.

**Figure 2.**
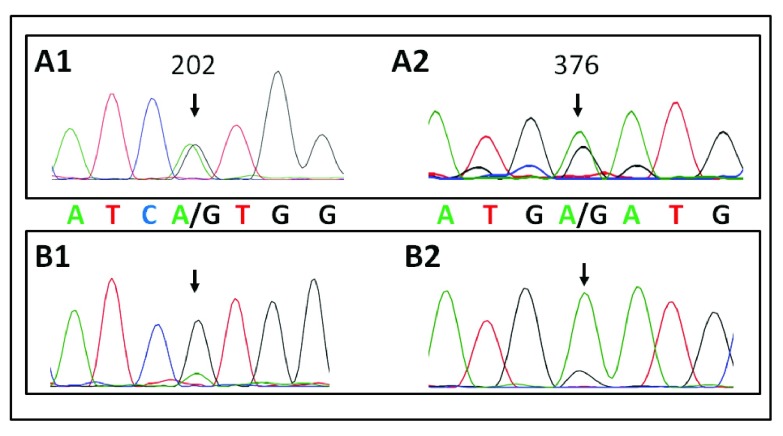
Patient CHOP 277.01. is heterozygous for G6PD B/A- and the B allele is predominantly expressed. A1 and A2 shows sequencing of genomic DNA around the 202 G->A and the 376 A->G mutation that lead to Val68Met and Asn126Asp changes in G6PD, respectively. Figures B1 and B2 show the cDNA sequence of the exact same mutations in the peripheral blood of the patient. The WT (B) allele is expressed in the majority of cells.

**Figure 3.**
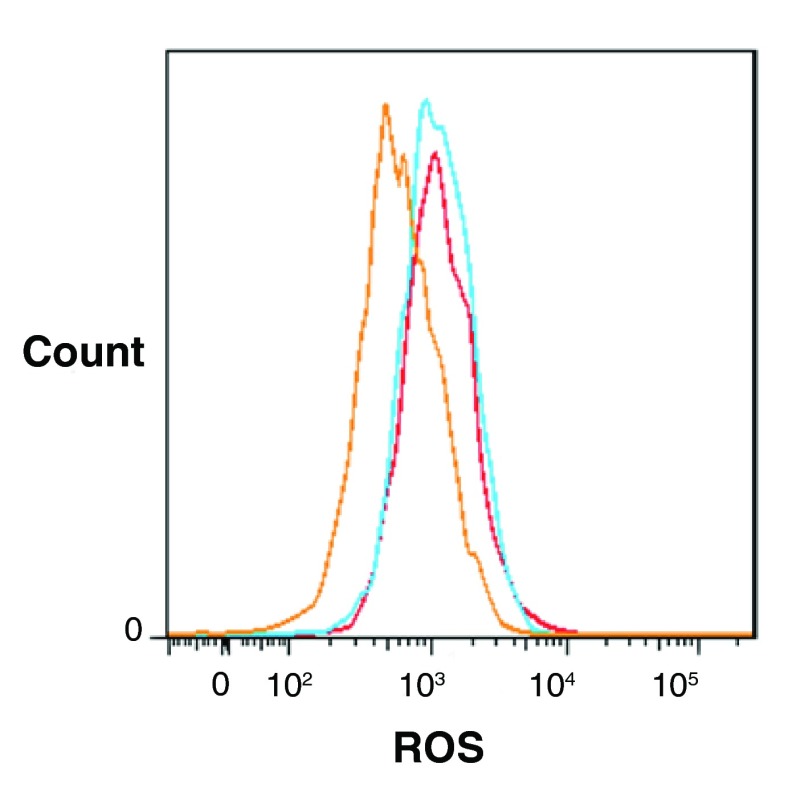
Flow cytometry analysis of the oxidative status of RBCs in the patient and a healthy donor. Peripheral blood mononuclear cells from the patient and a normal control were treated with the oxidation sensitive dye, CM-H2DCFDA, and the conversion to its oxidized fluorescent derivative assessed by flow cytometry. The fluorescence distribution histogram and the mean fluorescence channels (MFC) of each population derived from the normal control (orange) and the patient (blue for CD55 negative and red for CD55 positive cells) are shown. The figure shows a representative example of 4 normal controls that gave similar results.

This patient has a classic presentation of a patient whose blood cells mainly have a partial deficiency of GPI-linked proteins (PNH type II) with a significant delay in diagnosis relative infrequent hemolytic events and thrombotic complications. We were intrigued by the emergency physicians note that associated hemoglobinuria and clinical symptoms associated with hemolysis with the co-trimoxazole medication and family history of G6PD deficiency. We were therefore interested to find out whether she actually might have both, and whether her PNH might have been responsible for her G6PD deficiency and thereby explain the hemolysis precipitated by co-trimoxazole as noted by an obervant emergency physician. Sequencing of DNA from her granulocytes confirmed that she was heterozygous for *G6PD A*- having a *G6PD B* allele on one X-chromosome and a *G6PD A*- allele on the other. This finding raised the question as to whether the somatic *PIGA* mutation causing her PNH took place on the X-chromosome carrying the *B* or the *A- G6PD* gene. The flow cytometry data showed that the patient most likely had 2 PNH clones, a class II clone (partial deficiency) of about 86% and a class III clone (complete deficiency) of about 6%. The HUMARA assay, which measures X-inactivation, showed a single clone of about 90% ( Figure 1), suggesting that in both clones the mutation had taken place on the same X-chromosome. We hypothesized that the mutations in *PIGA* would have taken place on the chromosome carrying the *G6PD A-* allele since this would help explain the patient’s reaction to co-trimoxazole. To determine which *G6PD* allele was expressed in the PNH clone we sequenced cDNA from the patient’s granulocytes. The sequencing trace showed that the vast majority of expressed *G6PD* cDNA contained the wild type ( *G6PD B*) sequence at both nucleotides where it differs from *G6PD A-* ( Figure 2), leaving us to conclude that the *PIGA* mutations had taken place on the X-chromosome containing the *G6PD B* allele. This was confirmed at the protein level since the red blood cells lysed by acidified serum (the PNH cells) contained most of the G6PD activity while the residual cells did not contain detectable G6PD activity. While developing our hypothesis, which turned out to be incorrect, we also considered whether PNH/G6PDA- cells might have high levels of oxidative stress since both G6PD deficiency and PNH have been shown to be associated with elevated levels of reactive oxygen species ^11, 13^. We found that the patient’s red blood cells contained ROS levels that were significantly higher than those from healthy controls, though surprisingly we did not detect any difference in ROS between PNH (CD55-) and normal (CD55+) cells ( Figure 3).

## Discussion

PNH is a rare condition, having an incidence of about 1 in a million, so the co-incidental finding of a female with PNH and heterozygous for G6PD deficiency was an opportunity to observe the interaction between these 2 conditions which both involve red blood cell hemolysis mediated by X-linked genes. Notably the first demonstration that PNH was a clonal disease took advantage of a female PNH patient who was heterozygous for the electrophoretic variant *G6PD A* ^14^. In this patient both isozymes were present in a lysate of total red blood cells, but only one was present after acidified serum lysis, demonstrating clonality of the PNH cells.

In the case discussed here a female African American patient with PNH suffered episodes of hemolysis, often following treatment with Trimethoprim-Sulfamethoxazole (co-trimoxazole), one of the drugs that is known to cause hemolysis in G6PD patients ^15^. When it emerged that she was heterozygous for *G6PD A-* we hypothesized that her expanded PNH clones may be expressing only the G6PD A- protein, which would have explained the observation of the emergency physician. The hypothesis proved incorrect and the clone expressed the wild type *G6PD* allele. An alternative intriguing explanation for the co-trimoxazole associated hemolysis might be that complement activation on *G6PD A-* red cells exposed to co-trimoxazole might have triggered complement activation inducing the lysis of *G6PD B* PNH Type II red blood cells ^16^. naturally we cannot rule out that at the time the co-trimoxazole associated hemolysis was observed a *G6PD A-* PNH clone was more prevalent, since PNH patients, like the one described here, often have several PNH clones ^17^. Of course it is also possible that co-trimoxazole medication and hemolysis were coincidental and that hemolysis was primarily due to the urinary tract infection. Nevertheless the observation by an unbiased emergency physician and a rather bland urine sediment make us favor the first explanation. Finally, the association of PNH and G6PD deficiency make us also speculate that the combination of G6PD and PIGA deficiency confers a serious growth disadvantage and PNH clones in this situation are more likely to be G6PD wild type. There is no clear mechanism for this however as *G6PDA*- nucleated cells have similar enzyme activity to WT cells ^18^ – the deficiency becoming apparent in red blood cells, which do not synthesize new protein ^19^.

## Patient consent

Written informed consent for publication of their clinical details was obtained from the patient.
