# Supplementary material for: Case Report: Paroxysmal nocturnal hemoglobinuria in a woman heterozygous for G6PD A-
Source: F1000Res. 2014 Oct 21;3:194. Originally published 2014 Aug 13. [Version 2] doi: 10.12688/f1000research.4980.2 (PMC4335595; doi:10.12688/f1000research.4980.2)
Supplement: Peer review history

# Peer review: Referee response for version 2

**DOI:** 10.5256/f1000research.5927.r7539

**Published:** 2015-02-18

**Authors:** José M. Bautista

**Affiliations:** 1 Department of Biochemistry and Molecular Biology IV and Research Institute Hospital 12 de Octubre, Complutense University of Madrid, Madrid, Spain

This case report describes the rare concurrence of two haematological genetic disorders associated to chromosome X (PNH and G6PD deficiency) and provides a retrospective interpretation of the clinical development throughout the detailed and focused experimental analysis of the patient’s cells and the genetics associated to them. Following an appealing and reasonable hypothesis, the results obtained by Perdigones *et al.* offer an authoritative haematology lesson. Thus, the authors take the case report as a centre to discuss the biology of PNH clones and the potential mechanism of red cell lysis when two independent haemolysis-prone genes are associated in the same patient.

The originality and presentation of the case warrant the readers interest. The final version with the amendments made following the report from two previous reviewers resulted in a solid article worth reading for haematologists and geneticists.

I have read this submission. I believe that I have an appropriate level of expertise to confirm that it is of an acceptable scientific standard.

# Peer review: Referee response for version 2

**DOI:** 10.5256/f1000research.5927.r6632

**Published:** 2014-11-04

**Authors:** Anastasios Karadimitris

**Affiliations:** 1 Department of Haematology, Imperial College Healthcare NHS Trust and Imperial College, London, UK

I have read this submission. I believe that I have an appropriate level of expertise to confirm that it is of an acceptable scientific standard.

# Peer review: Referee response for version 1

**DOI:** 10.5256/f1000research.5317.r6022

**Published:** 2014-09-04

**Authors:** Anastasios Karadimitris

**Affiliations:** 1 Department of Haematology, Imperial College Healthcare NHS Trust and Imperial College, London, UK

Perdigones and colleagues describe the rare co-occurence in the same patient of two X-linked disorders, one acquired the other inherited, both causing intravascular haemolysis.

However how the interaction of the two disorders led to the clinical episode described in the case report is not clear because the temporal analysis of the clinical events and the associated laboratory tests are not presented in sufficient detail.

Charting clinical events, labs and therapeutic interventions might make association of the heamolytic episodes with co-trimoxazole or intercurrent infection clearer.

It appears that the majority of the PNH clone (in both granulocytes and red cells) is type II, i.e., only partially deficient of GPI. One assumes that this picture was obtained after the haemolytic attack. If so, it could be that type III, i.e., severely deficient RBC were indeed G6PD-deficient. Was flow-cytometry performed after Eculizumab treatment? Eculizumab would protect type III RBC from lysis and thus would allow re-assessment of G6PD activity.

**Minor points**

- Was anti-CD55 or –CD59 was used for flow analysis? The authors state anti-CD55 in methods but describe anti-CD59 in results.
- Normal ranges of lab tests need to be provided
- ‘Another factor was that treatment with eculizumab, by inhibiting lysis of PNH red cells may have led to a higher level of PNH (and concomitantly G6PD deficient) red cells than would be present in untreated patients.’ This statement is rather irrelevant because eculizumab was started after treatment with co-trimoxazole.
- Need to use Greek characters appropriately, i.e., ul should be ml 

I have read this submission. I believe that I have an appropriate level of expertise to confirm that it is of an acceptable scientific standard, however I have significant reservations, as outlined above.

# Response

**Published:** 2014-10-15

**Authors:** Philip Mason

**Affiliations:** Leonard and Madlyn Abramson Pediatric Research Center, The Children's Hospital of Philadelphia, USA

*It appears that the majority of the PNH clone (in both granulocytes and red cells) is type II, i.e., only partially deficient of GPI. One assumes that this picture was obtained after the haemolytic attack. If so, it could be that type III, i.e., severely deficient RBC were indeed G6PD-deficient. Was flow-cytometry performed after Eculizumab treatment? Eculizumab would protect type III RBC from lysis and thus would allow re-assessment of G6PD activity.*

We agree with this comment. Of course it is possible that at the time of the observation of association of *co-trimoxazole* associated hemolysis a different PNH clone was more prevalent than when analyzed for G6PD deficiency. We included this possibility in our discussion. The most recent flow cytometry was performed when the patient was on eculizimab.

**Minor points**

- *Was anti-CD55 or –CD59 was used for flow analysis? The authors state anti-CD55 in methods but describe anti-CD59 in results. The diagnosis for PNH was performed in a CLIA approved clinical laboratory of the hospital.*
  We present the results for CD59. CD55 was also tested however the discrimination between the three populations is more difficult.
- * Normal ranges of lab tests need to be provided.*
  We have included normal ranges for laboratory tests in parentheses after the patient’s values.
- *Another factor was that treatment with eculizumab, by inhibiting lysis of PNH red cells may have led to a higher level of PNH (and concomitantly G6PD deficient) red cells than would be present in untreated patients.’ This statement is rather irrelevant because eculizumab was started after treatment with co-trimoxazole.*
  We think this statement is valid because treatment with eculizimab increases the level of PIGA deficient red cells. On our hypothesis that the G6PDA- allele was linked with PIGA- then PIGA-,G6PDA- red cells would increase. Our hypothesis however was not correct.
- *Need to use Greek characters appropriately, i.e., ul should be ml *
  We have corrected our incorrect use of Greek characters.

# Peer review: Referee response for version 1

**DOI:** 10.5256/f1000research.5317.r5816

**Published:** 2014-08-28

**Authors:** Rosario Notaro

**Affiliations:** 1 Laboratory of Genetics and Gene Transfer, Core Research Laboratory, Istituto Toscano Tumori, Florence, Italy

Perdigones and collaborators report an interesting clinical case about the association of PNH and G6PD deficiency. However, the timing of the clinical history is not clear and the report is scanty of some relevant clinical/laboratory data.

**Major scientific points.**

1. The Authors should provide the exact timing of entire clinical history: diagnosis; infective episode resulting in the prescription of *trimethoprim-sulfamethoxazole -co-trimoxazole-* (which dose?); start and stop of *co-trimoxazole*; “dark urine episode” associated with *co-trimoxazole*; start of *eculizumab*; time of biological studies.
2. It is extremely important that the Authors provide more details about the timing and the features of the hemolytic attack apparently associated with *co-trimoxazole*: how long after the start of *co-trimoxazole* treatment the patient experienced the “dark urine episode”? There are any objective data at the time of this “dark urine episode” or the episode has been just self-reported? At the time of this “dark urine episode” there were any signs/symptoms of an ongoing infective condition?
3. The Authors provide clinical/laboratory details only at one time point, that seems be after the start of eculizumab (how long after?). They should provide such clinical/laboratory details at time of diagnosis, at the time of the co-trimoxazole associated “hemolytic attack”, at start of eculizumab treatment, etc.: blood count, absolute reticulocyte count, LDH levels (providing the normal range), flow citometry, etc.
4. In PNH patients the presence of red blood cells with partial deficiency of GPI-linked molecules is relatively common. However, the presence of granulocyte/monocyte with partial deficiency of GPI-linked molecules is uncommon: thus, it would be interesting to show the dot plot of the “CD59/lineage marker” analysis of granulocytes and monocytes of this patient.
5. The Authors, at variance with their starting hypothesis, have clearly proven that in this patient PNH cells express the wild type G6PD B. They provide 2 possible explanations for the *co-trimoxazole*-associated “hemolytic crisis” observed in this patient. These hypotheses are interesting but their probability is very low. I suggest that the Authors should discuss a much more likely explanation: this “hemolytic crisis” was just due to the infective condition that led to the prescription of *co-trimoxazole*.

**Minor points**

1. In the Introduction the Authors report the classical classification of PNH cells as Type III (completely deficient in GPI anchored proteins), Type II (partially deficient) and Type I (normal display of GPI-linked proteins). However, in the Results they write about “ *class I clone (partial deficiency) … and class II clone (complete deficiency) *“:  this is extremely confusing.
2. In the Results the 2 sentences (from “ *We hypothesized that …*” to “ *…red cells than would be present in untreated patients.*”) seem to suggest that the relative expansion of the “PNH (and concomitantly G6PD deficient) red cells” following *eculizumab* treatment could have played a role in the *co-trimoxazole* associated hemolytic attack. The Authors should rephrase these sentences since *eculizumab* has been started after the *co-trimoxazole* associated hemolytic attack.
3. *Trimethoprim-sulfamethoxazole* (co-trimoxazole) is a generic drug name thus it should not be capitalized. In the Results, “ *eculizumab*” should not be capitalized.
4. The brand name *Bactrim* should be replaced with the generic drug name.
5. The levels of ROS in the patient red cells should be compared with a group of healthy controls: the comparison with only one control does not allow any conclusion.
6. The Authors should report the concentration of DCF used for ROS detection.

I have read this submission. I believe that I have an appropriate level of expertise to confirm that it is of an acceptable scientific standard, however I have significant reservations, as outlined above.

# Response

**Published:** 2014-10-15

**Authors:** Philip Mason

**Affiliations:** Leonard and Madlyn Abramson Pediatric Research Center, The Children's Hospital of Philadelphia, USA

*Perdigones and collaborators report an interesting clinical case about the association of PNH and G6PD deficiency. However, the timing of the clinical history is not clear and the report is scanty of some relevant clinical/laboratory data.*

***Major scientific points.***

1. *The Authors should provide the exact timing of entire clinical history: diagnosis; infective episode resulting in the prescription of trimethoprim-sulfamethoxazole -co-trimoxazole- (which dose?); start and stop of co-trimoxazole; “dark urine episode” associated with co-trimoxazole; start of eculizumab; time of biological studies.*
  In our revised manuscript we include a fuller, more detailed case history. Of note is the fact that the patient presented to the specialty clinic with a probably 6 year history of PNH and that most of the patients past history relies on records and notes. Here we demonstrate that the patient has indeed both conditions PNH and G6PD deficiency. With these results in hand we try to explain the patient’s history and clinical observations. It was not our intention to prove that indeed the hemolysis described by the emergency physician after *co-trimoxazole* was indeed triggered by the drug, but rather to evaluate and discuss the possibility as the patient carries on her chart the diagnosis of *co-trimoxazole* hypersensitity due to this incident. We try to make this clearer in the case description. We hope this will answer the criticism of the reviewer.
2. *It is extremely important that the Authors provide more details about the timing and the features of the hemolytic attack apparently associated with co-trimoxazole: how long after the start of co-trimoxazoletreatment the patient experienced the “dark urine episode”? There are any objective data at the time of this “dark urine episode” or the episode has been just self-reported? At the time of this “dark urine episode” there were any signs/symptoms of an ongoing infective condition?*
  The discussion above and the revised version provides many more details.
3. *The Authors provide clinical/laboratory details only at one time point, that seems be after the start of eculizumab (how long after?). They should provide such clinical/laboratory details at time of diagnosis, at the time of the co-trimoxazole associated “hemolytic attack”, at start of eculizumab treatment, etc.: blood count, absolute reticulocyte count, LDH levels (providing the normal range), flow citometry, etc.*
  We have provided all the history we can obtain in the revised version.
4. *In PNH patients the presence of red blood cells with partial deficiency of GPI-linked molecules is relatively common. However, the presence of granulocyte/monocyte with partial deficiency of GPI-linked molecules is uncommon: thus, it would be interesting to show the dot plot of the “CD59/lineage marker” analysis of granulocytes and monocytes of this patient.*
  The diagnosis of PNH and the subtype analysis of PNH granulocytes was performed in a CLIA approved clinical laboratory, and their test results are reported here. The individual dot blots are not available to us. However the senior author who was involved in the setup and quality assessment of flow-cytometric PNH testing at this institution fully trusts their analysis. We agree with the reviewer that partial GPI-anchor deficiency in granulocytes is less frequently observed and very much depends on the underlying mutation and the antibody chosen for the analysis.
5. The Authors, at variance with their starting hypothesis, have clearly proven that in this patient PNH cells express the wild type G6PD B. They provide 2 possible explanations for the *co-trimoxazole*-associated “hemolytic crisis” observed in this patient. These hypotheses are interesting but their probability is very low. I suggest that the Authors should discuss a much more likely explanation: this “hemolytic crisis” was just due to the infective condition that led to the prescription of *co-trimoxazole*.
*We agree that this is a possible alternative explanation and have added this in the discussion however considering the rather bland urine sediment we actually favor the other two possible explanations.*

***Minor points***

1. *In the Introduction the Authors report the classical classification of PNH cells as Type III (completely deficient in GPI anchored proteins), Type II (partially deficient) and Type I (normal display of GPI-linked proteins). However, in the Results they write about “class I clone (partial deficiency) … and class II clone (complete deficiency) “:  this is extremely confusing.*
  This has been corrected
2. *In the Results the 2 sentences (from “We hypothesized that …” to “…red cells than would be present in untreated patients.”) seem to suggest that the relative expansion of the “PNH (and concomitantly G6PD deficient) red cells” following eculizumab treatment could have played a role in the co-trimoxazole associated hemolytic attack. The Authors should rephrase these sentences since eculizumab has been started after the co-trimoxazole associated hemolytic attack.*
  The timing of the eculizimab treatment and the hemolysis are given in the detailed case report that we now provide.
3. *Trimethoprim-sulfamethoxazole (co-trimoxazole) is a generic drug name thus it should not be capitalized. In the Results, “eculizumab” should not be capitalized.*
  We have corrected this.
4. *The brand name *Bactrim** *should be replaced with the generic drug name.*
  We have corrected this.
5. *The levels of ROS in the patient red cells should be compared with a group of healthy controls: the comparison with only one control does not allow any conclusion.*
  The experiment was carried out with several controls and it confirms the results of Amer *et al.*, cited as our ref 14 that ROS are elevated in PNH. Our point is a small one, that we might expect a further elevation if we compare PNH and normal cells from this patient, if indeed PIGA- is linked with G6PDA-. We didn’t find this. We include the sentence “The figure shows a representative example of 4 normal controls that gave similar results” to clarify this.
6. *The Authors should report the concentration of DCF used for ROS detection.*
  We have done this.
